# Presence of The NLRP3 Inflammasome Components in Semen of Varicocele Patients

**DOI:** 10.22074/ijfs.2020.5734

**Published:** 2020-02-25

**Authors:** Maryam Baazm, Ali Asghar Ghafarizadeh, Ali Reza Noshad Kamran, Cordian Beyer, Adib Zendedel

**Affiliations:** 1 Department of Anatomy, School of Medicine, Arak University of Medical Sciences, Arak, Iran; 2 Infertility Center of ACECR, Arak, Iran; 3 Department of Urology, School of Medicine, Arak University of Medical Sciences, Arak, Iran; 4 Institute of Neuroanatomy, RWTH Aachen University, 52074 Aachen, Germany; 5 Department of Anatomy, School of Medicine, Tehran University of Medical Sciences, Tehran, Iran

**Keywords:** Inflammasome, Semen, Varicocele

## Abstract

**Background:**

Varicocele is a common cause of male infertility with multifactorial etiology. Inflammation is a characteristic pathological event that occurs in the testis tissue following the varicocele. The aim of this study was to investigate expression of nod-like receptor family, pyrin domain containing 3 (NLRP3) inflammasome components and cytokines in semen of varicocele and control subjects.

**Materials and Methods:**

In this case-control study, seminal plasma was collected from 32 varicocele patients (with grades 2 and 3) and 20 fertile men as control group. Semen analysis was performed in all subjects. Concentrations of interleukin-1b (IL-1b), IL-18 and caspase-1 in seminal plasma were measured by enzyme-linked immunosorbent assay (ELISA). Apoptosis-associated speck-like protein containing a caspase activation and recruitment domain, in addition to NALP3 were identified in seminal plasma by Western blot. Statistical significance between the mean values was determined by student’s t test.

**Results:**

According to our data, the level of IL-1b was significantly (P=0.03) increased in the seminal plasma of varicocele patients, compared to the control subjects. We analyzed amount of IL-18 in the both groups. The level of this interleukin was markedly (P=0.002) decreased in varicocele patients. No change was observed in the level of caspase-1 in both groups. Western blot analysis revealed that apoptosis associated speck-like protein (ASC, P=0.0002) and NLRP3 (P=0.005) were significantly elevated in the semen of varicocele patients.

**Conclusion:**

This study provides the first evidence of activation of NLRP3 components in semen of men with varicocele.

## Introduction

Varicocele is one of the most common causes of male infertility. Approximately, 15% of healthy men and 40% of infertile men suffer from varicocele (1). This gonadal disease is defined as a pathological dilation of testicular venous plexus (pampiniform plexus) and it is associated with pathological problems in the testicular tissue. Typically, it occurs on the left side (2). Varicocele could interfere with normal spermatogenesis which leads to production of abnormal spermatozoa (3, 4).

In varicocele disease, heat stress induces undesirable adverse effects on testis tissue, such as spermatogenesis impairment, increase in production of reactive oxygen species (ROS) and apoptosis (5). On the other hand, the stasis of venous blood in the dilated pampiniform plexus impairs arterial blood flow and restricts oxygen supply necessary for testis tissue which can lead to the testicular hypoxia (6). It is believed that hypoxia signaling pathway is responsible for pathogenesis of varicocele (7). However, there are studies suggesting that varicocele stimulates pro-inflammatory and inflammatory cytokines release, such as interleukin-1 (IL-1), IL-6, IL-8 and tumor necrosis factor-alpha (TNF-α) (8-11).

Inflammation is an immune response to pathological events, such as bacterial/viral infection and tissue damage to protect other cells from injury (12). Inflammation can be triggered by necrosis or pyroptosis. The latter is involved in receptor-mediated sensing of pathogens, cell fragments, ATP as well as the activation of intracellular multiprotein complex named inflammasome. Inflammasomes exist in different subtypes and represent a complex of proteins assembly. They activate caspase-1 which, in turn, promotes maturation of pro IL-1 and IL-18 into their active forms. Among the different types of inflammasome (NLRP1, NLRP2, NLRP3 and AIM), the role and regulation of Nodlike receptor family, pyrin domain containing 3 (NALP3) inflammasome is well studied. The structure of NLRP3 consists of three components: a central nucleotide-binding and oligomerization (NACHT) domain, a ligand-sensing leucine-rich repeat domain (LRRs) and a pyrin (PYD) domain. NLRP3 activation leads to the oligomerization of apoptosis associated speck-like protein (ASC) which contains a caspase activation and recruitment domain (CARD). Apoptosis associated speck-like protein (ASC) interacts with the CARD of pro-caspase-1 and converts it to the active form. Activated caspase-1 then proceeds to generate active form of IL-1b and IL-18 from the immature forms (13).

In testis of rodents and primates, Sertoli cell is responsible for NLRP3 expression and it is believed that alteration in NLRP3 expression might impair fertility (14). Increased level of *NLRP3* mRNA in rat testis tissue was induced seven days after spinal cord injury (SCI) (15). In addition, overexpression of the NLRP3 components (ASC, caspase-1, IL-1β and IL-18) were identified in the seminal plasma of patients with SCI, while the previous studies showed that inflammasomes are responsible for abnormal semen quality in these patients (16). Recently we showed NLRP3 complex expressed in testis tissue of varicocelized rats and resveratrol as an antioxidant could decrease its expression (17). Due to this finding and presence of some pro-inflammatory cytokines during the course of varicocele (10, 11), we hypothesized that NLRP3 inflammasome components might be present in semen of varicocele patients and inflamatory events are involved in the pathogenesis of varicocele in addition to the hypoxia pathway. Therefore, the aim of this study was to investigate presence of NLRP3 complex in seminal plasma of varicocele patients.

## Materials and Methods

### Study design

This study was performed from December 2017 to September 2018. Sample size was calculated by the following formula:

$$n\geq \frac{{\left(Z_{\frac{\alpha }{2}}+Z_{\beta }\right)}^{2}\sigma^{2}}{\varepsilon^{2}}$$

where type one (α) and type two errors (β) were 0.05 and 0.20 (power ¼; 85%), respectively according to previous studies (7, 18). Based on this, we needed at least 20 subjects in each group. Semen samples were collected from 32 men with varicocele and 20 age-matched control subjects attending the Iranian Academic Centre for Education, Culture and Research (ACECR). All control subjects had no history of infertility with normal sperm analysis who volunteered to take part in this research. The mean ± standard error of the mean (SEM) age of varicocele patients and control subjects were 27 ± 2.1 years and 26 ± 1.8 years, respectively. The patients had palpable varicocele (at grades 2 and 3) with a clear history of infertility (for 2-3 years). Infertility is defined as inability to have children after at least one year of unprotected intercourse (19). The study was approved by the Ethics Committee of Arak University of Medical Sciences (code: 93-175-10; Arak, Iran) and all patients signed the informed consent for this study.

### Seminal collection

Semen samples were collected from varicocele and control subjects by masturbation after at least 48 hours of sexual abstinence. After liquefaction for 30 minutes, semen parameters including volume, pH, concentration, morphology and motility were analyzed according to the World Health Organization criteria (20). Analysis of sperm concentration was performed with a Neubar chamber on two separate preparations of the semen sample (dilution 1:20 in Ringer’s solution). A standard volume of semen (approximately 10 µl) was placed onto a glass slide and covered by the cover slide, then 200 spermatozoa were assessed under a light microscope (×400 magnification) for the percentage of sperm motility. To evaluate sperm morphology Papanicolaou staining was used and one hundred sperm from different fields were counted to determine the morphological abnormalities (21).

### Seminal plasma preparation

Semen samples were centrifuged at 1000 xg for 15 minutes at room temperature. The supernatant was then collected and stored at -70°C for further analysis (16).

### ELISA analysis

Concentrations of the mature IL-1β, IL-18 and caspase-1 (all from Abcam, USA) were measured by an enzymelinked immunosorbent assay (ELISA) kit following the manufacturer’s protocol. Seminal plasma samples were thawed at room temperature and placed in plates precoated with a specific monoclonal antibody for each of IL-1b, IL-18 or caspase-1. Each sample was duplicately assayed.

### Western blot

Seminal plasma samples were thawed at room temperature. One microliter of seminal plasma from each subject was mixed with loading buffer (containing a final concentration of 50 mmol/l Tris-HCl pH=7.0, 2% sodium dodecyl sulfate, 10% glycerol, 5% b-mercaptoethanol and 0.002% bromophenol blue), and heated at 95°C for 10 minutes. Protein concentrations were determined using the BCA™ Protein Assay Kit (Pierce, Germany) according to the manufacturer’s protocol. The same amount of protein samples was loaded, separated on 8-12% (v/v) discontinuous sodium dodecyl sulfate polyacrylamide gel electrophoresis (SDS-PAGE) and transferred onto a polyvinylidenefluoride (PVDF) membrane (Roche, Germany). After blocking with 5% skimmed milk in Tris-buffered saline containing 0.05% Tween 20 (TBS-T) for 1 hour at room temperature, PVDF membranes were incubated with anti-ASC antisera (Santa Cruz, USA, diluted 1:1000) or anti-NLRP3 antisera (Bioss, USA, diluted 1:1000) overnight at 4oC. After washing with TBS-T, membranes were incubated with a peroxidase-conjugated goat anti-rabbit (BioRad, USA, diluted 1:500) secondary antibody for 2 hours at room temperature. Visualization was performed using the enhanced chemiluminescence method (ECL plus, Pierce Scientific, USA) according to the manufacturer’s protocol. For densitometric quantification, intensity of the specific bands was normalized to β-actin (Bioss, USA, diluted 1:1000) in the same blot using Image J software (free Java software provided by the National Institute of Health; Bethesda, USA) (22).

### Statistical analysis

The results are expressed as means ± standard errors (SE). The Shaprio-wilktest was used to determine normal distribution. Independent sample t test was applied to check the matched factor between the case and control groups. Statistical significance between the mean values was determined by paired t test and P≤0.05 was considered statistically significant.

## Results

In this study, we analyzed relationship of sperm parameters with NLRP3, ASC, IL-18 and caspase-1 in both control and varicocele groups. We could not find any correlation in our study.

### Semen quality is lower in varicocele patients compared to the control subjects

The semen volume (5.1 ± 1.8 ml in control vs. 4.06 ± 1.5 in varicocele) and pH (7.8 ± 0.2 in control vs. 7.7 ± 0.1 in varicocele) were not significantly different between the control and varicocele subjects. The median sperm concentration in the varicocele group (44 million/ml) was significantly lower than the control group (97 million/ml, P=0.0001).The median sperm total motility was equal in the both groups (61% in control vs. 55% in varicocele) while the sperm progressive motility in varicocele patients showed a significant decrease (P=0.0003) compared to the control subjects. In addition, varicocele patients had more abnormal sperm (especially abnormal head) morphology compared to the control group (99% in varicocele vs. 80% in control).

### Alteration in inflammatory cytokine levels in varicocele patients

**Fig 1:**
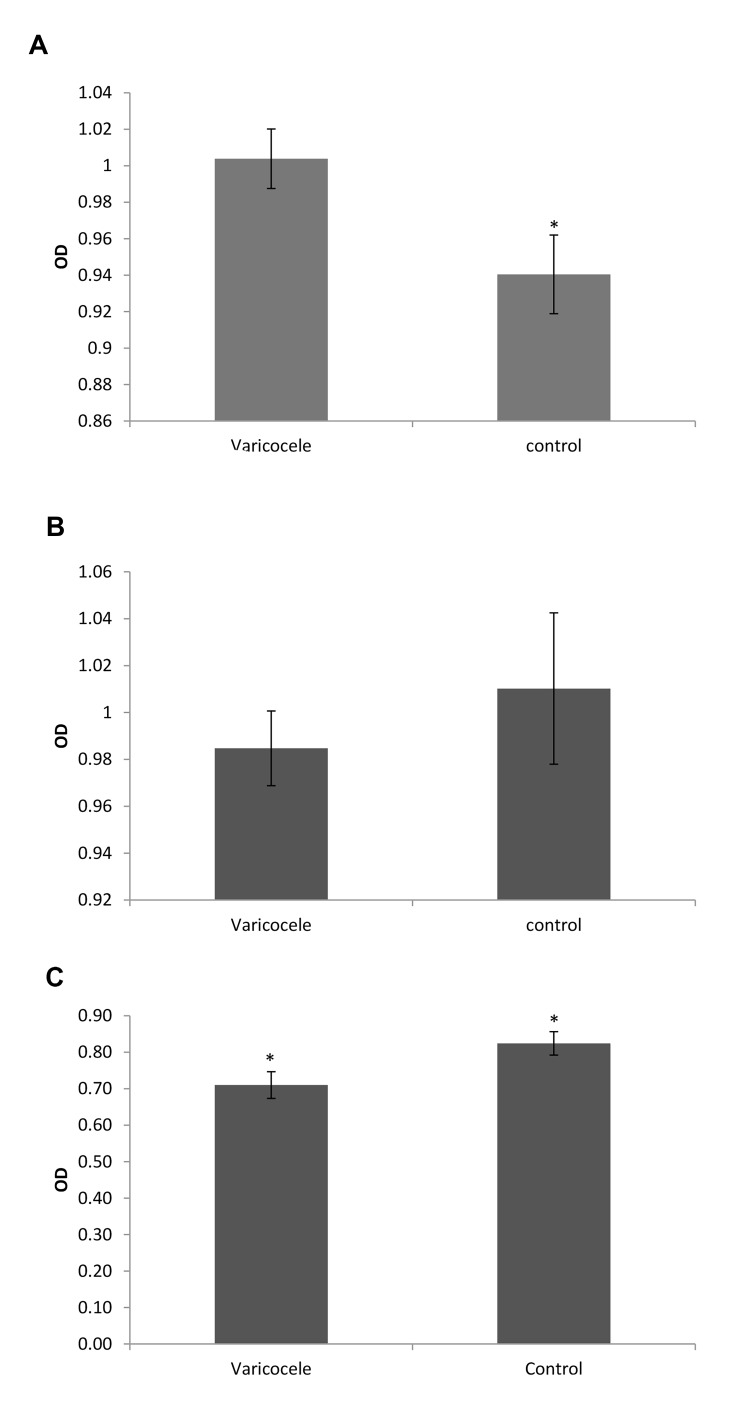
Measurement of the inflammatory cytokines in seminal plasma of varicocele patients using ELISA. **A.** Note the significant increase of IL-1ß protein levels in varicocele patients compared to the control subjects. **B.** Caspase-1 protein level did not reveal significant change between two groups. **C.** Note the significant decline of IL-18 protein levels in the varicocele group compared to the controls. OD; Optic densitometry and *; P≤0.05 compared to the controls.

The levels of IL-1β, IL-18 and caspase-1 were investigated in seminal plasma by ELISA. Our data showed that IL-1β was significantly increased (P=0.03) in seminal plasma of varicocele patients in comparison with control subjects [optic densitometry (OD)=1 ± 0.016 vs. 0.94 ± 0.021, respectively; Fig.1A]. In these patients, concentration of caspase-1 showed no obvious change (Fig .1B), while seminal plasma concentration of IL- 18 revealed a small but significant (P=0.002) decline in varicocele versus controls (OD=0.71 ± 0.036 vs. 0.82 ± 0.032, Fig .1C).

### Inflammatory NLRP3 and ASC protein are elevated in seminal plasma of varicocele subjects

**Fig 2:**
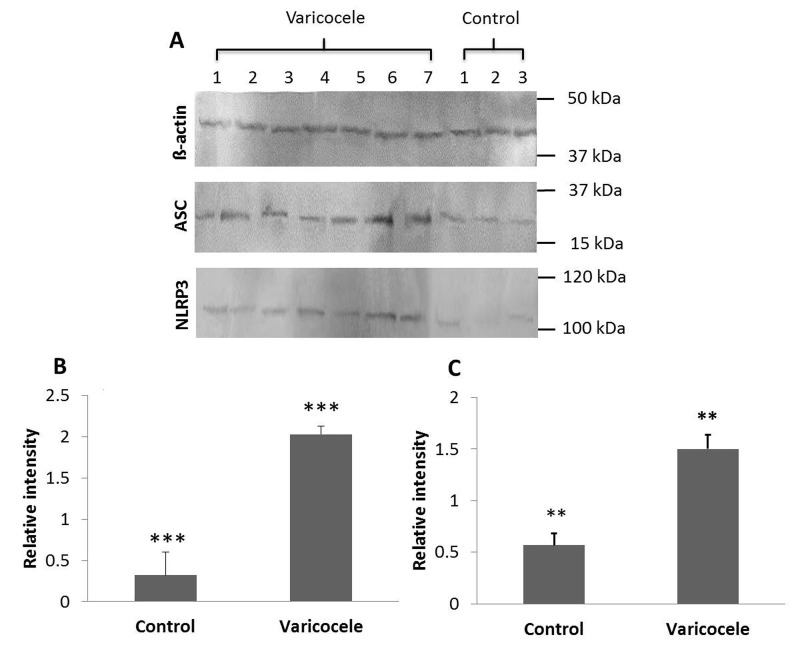
Analysis of inflammasome components in seminal plasma of varicocele patients by Western blotting and subsequent measurement of optical densities of immune-labelled bands. **A.** Respresentative Western blots with seven samples of varicocele patients and three control subjects. Note the increased intensities of NLRP3 and ASC in all varicocele patients. Quantification of the respective band intensities for **B.** ASC and **C.** NLRP3 given as relative intensities compared to ß-actin bands. **; P≤0.01 and ***; P≤0.001 compared to controls.

To investigate whether inflammasome components are expressed and changed in seminal plasma of varicocele patients, we quantified respectively ASC and NLRP3 protein levels by Western blot. NLRP3 (P=0.005) and ASC (P=0.0002) protein levels were significantly elevated in varicocele patients versus control subjects (relative intensity of ASC was 2.02 ± 0.09 vs. 0.32 ± 0.28 and relative intensity of NLRP3 was 1.5 ± 0.13 vs. 0.56 ± 0.1, respectively, Fig. 2 A-C).

## Discussion

In this study, quality of semen in all varicocele patients was lower than the control group. Varicocele patients had abnormal semen quality (23) which might be because of high ROS level (18), germ cell apoptosis and release of the inflammatory cytokines (5). Although previous studies suggesting the inflammatory cytokines are present in semen of men with varicocele, the inflammasome signalling mechanism in varicocele has not been previously tested. The current study indicates, for the first time, that inflammasome components ASC and NLRP3 are present in semen of varicocele patients.

In this study our results showed that ASC and NLRP3 levels in semen of varicocele subjects were significantly elevated compared to the control subjects. Additionally, concentration of IL-1β was higher in varicocele versus control subjects, whereas IL-18 was decreased in seminal plasma of varicocele patients and caspase-1 was not changed. In addition, we could not find any significant correlation between sperm parameters and NLRP3 inflammasome components.

Sahin et al. (24) acclaimed that amount of proinflammatory cytokines such as IL-1α and IL-1β were increased 11 and 13 weeks after the induction of varicocele in rats. They showed that during progression of the disease, IL-1α was expressed in round spermatids, spermatogonia, primary spermatocytes, Sertoli and Leydig cells, while IL-1β was found only in spermatogonia, Leydig and Sertoli cells. Concentration of the caspase-1 which is involved in IL-1β and IL-18 maturation (13), was equal in testis tissue of the both varicocele and normal subjects (25). During varicocele condition, an excessive release of nitric oxide (NO) into the seminal plasma occurs and this is believed to be a reason of low motility in subfertile patients with varicocele (26, 27). Kim et al. (28) revealed that NO production inhibits caspase-1 activity and subsequently inflammatory responses. However, NO accumulation could damage tissue itself. In the current study, absence of any obvious changes of caspase-1 protein level might therefore reflect neither the enzyme activity nor the influence of an excessive NO production in varicocele patients.

In this study, we expected that IL-18 level would be increased in varicocele subjects (29), while it was decreased. The difference between our results and the investigation performed by Zeinali et al. (29) could be related to different numbers and ages of the studied samples.

Western blot analysis showed high level of NLRP3 and ASC protein expressions in seminal plasma of varicocele patients. In our previuos work, we showed high levels of ASC, NLRP3 and caspase 1 expression in the testis tissue of varicocele-induced rats, three months after surgery (17).

Novelty is the strength of this study, as this is the first evidence for the existence and presence of the NLRP3 inflammasome in seminal plasma of varicocele patients. Thus, it highlights the importance of anti-inflammasome therapies to improve the fertility rate in varicocele patients. However, a limitation is about the control subjects. It was better to choose fertile varicocele subjects as control group. The other weak point is that the immunohistochemistry was not done in this study to localize this complex. 

## Conclusion

Findings obtained from this study suggest that NLRP3 activation occurs in varicocele and it might be responsible for pathological procedure occurring in varicocele patients. Details of the NLRP3 inflammasome activation process has not been clarified yet. At present, it is not clear whether NLRP3 is causally related to the onset of varicocele or the result of pathological damages appearing in the course of this gonadal disease. Further study is underway to determine time course of activation of inflammasome in varicocele disease.
